# Health students’ knowledge about healthy eating and factors associated with the university environment

**DOI:** 10.17843/rpmesp.2022.394.11349

**Published:** 2022-12-29

**Authors:** Isabelle Cerqueira Sousa, Ana Maria Fontenelle Catrib, Natasha Teixeira Medeiros, Carla Christina Pereira da Silva Godinho, Antonio Augusto Ferreira Carioca, Gabriela Pessoa Marinho Holanda, Ilana Nogueira Bezerra, Ana Paula Vasconcellos Abdon

**Affiliations:** 1 Postgraduate Program in Collective Health (PPGSC), University of Fortaleza (UNIFOR), Fortaleza, Ceará, Brasil University of Fortaleza Postgraduate Program in Collective Health (PPGSC) University of Fortaleza (UNIFOR) Fortaleza Ceará Brazil; 2 Federal del Delta do Parnaíba University (UFDPar), Parnaíba, Piauí, Brasil Federal del Delta do Parnaíba University Federal del Delta do Parnaíba University (UFDPar) Parnaíba Piauí Brazil; 3 University of Fortaleza (UNIFOR), Fortaleza, Ceará, Brasil University of Fortaleza University of Fortaleza (UNIFOR) Fortaleza Ceará Brazil; 4 Postgraduate Program in Collective Health, Universidad Estatal de Ceará (UECE), Fortaleza, Ceará, Brasil Universidade Estadual do Ceará Postgraduate Program in Collective Health Universidad Estatal de Ceará (UECE) Fortaleza Ceará Brazil

**Keywords:** Healthy Diet, Universities, Health Promotion

## Abstract

**Objective.:**

To assess the healthy eating (HE) knowledge of health students and the factors associated with the university environment.

**Materials and methods.:**

This was a cross-sectional study of 512 university students (≥ 18 years) enrolled in nine undergraduate health careers. It was conducted from April to November 2017. The Instrument for Assessment of Health Promotion in Universities and the International Physical Activity Questionnaire were used. In addition, we measured weight, height and waist circumference. Bivariate and multivariate analyses were carried out with SPSS version 23.0.

**Results.:**

We found that most university students from the nine health careers had insufficient knowledge about healthy eating (71.9%; n=368). However, the highest proportion of students with sufficient knowledge was found in the career of nutrition (15.3%; n=22), followed by those in the physical education career (12.5%; n=18). The lowest percentage of students with sufficient knowledge was found in the career of medicine (8.3%; n=12). Multivariate analysis showed that sufficient knowledge about healthy eating was related to participation in healthy eating activities (p=0.012; PR=1.94), participation in activities addressing self-esteem and self-knowledge (p=0.046; PR=0.59) and being overweight (p=0.036; PR=1.53).

**Conclusion.:**

A low percentage of health students had sufficient knowledge about healthy eating. However, participation in healthy eating, self-esteem and self-knowledge activities at the university managed to improve the level of knowledge. We recommend the development of university projects that include the psychological, food and body triad, thus involving all health careers, with the aim of improving the health and quality of life of university students.

## INTRODUCTION

Adequate and healthy food is a human right, which must be aligned with the biological and social aspects of the individual, according to his or her special dietary needs, and based on the region’s cultural background. It should be available, affordable, harmonious in quantity and quality, in accordance with the principles of variety, balance, moderation and pleasure, in addition to being based on adequate and sustainable production practices ^1^.

The Family Budget Survey (*Pesquisa de Orçamentos Familiares* - POF) analyzed the food consumption of the Brazilian adult population between 2017 and 2018 and found that there was a reduction, compared to the POF conducted in the previous decade, in the consumption of rice (OR=0.38 for men; OR=0.47 for women) and beans (OR=0.32 for men; 0.41 for women). These two foods represent a perfect combination of amino acids, being part of the traditional diet in Brazilian culture. In addition, consumption of sandwiches increased (OR=3.01 for men; OR=3.01 for women) in all regions of the country and in all social classes, while the consumption of fruits decreased (OR=0.52 for men; OR=0.63 for women) ^2^.

The eating habits of university students change when they enter college. The daily routine can often have a negative influence on the eating habits of students, since most of the time they eat away from home, causing students to rely on commercial establishments at or near the university ^3^. In addition, only 7% of university students plan the timing of their meals throughout the day ^4^, although this is one of the ten steps established in the Brazilian Population Food Guide for healthy eating ^1^. 

The university food environment may be a factor involved in the exposure to chronic noncommunicable diseases in young adults, as unhealthy foods and beverages are more accessible. According to a systematic review, universities in the United States and the European Union have noticed the importance of this issue and have implemented interventions to improve the food environment. Some of these interventions focused on guidelines for food labeling, increasing the availability of healthy foods, and decreasing the portion size of unhealthy foods ^5^. These actions show the capacity of higher education institutions to work in favor of health promotion, especially dietary health promotion.

It is also evident that pursuing a health care career is not a protective factor for having a healthier diet, because unhealthy eating behaviors are frequent in this field, with high consumption of fast food, sweets, soft drinks, and alcoholic beverages, as well as low consumption of fruits, vegetables, fish, whole grains, and legumes. Therefore, regardless of the undergraduate course, food consumption by university students has been characterized as inadequate ^6^^,^^7^^,^^8^^,^^9^.

In addition, it is possible to evidence the increasing consumption of industrialized products, especially among adults and university students. Poor understanding of product labels or even not reading them is also an important fact to consider, therefore it is necessary to have a critical look at food choices ^7^. 

The fundamental actions aimed at the promotion and protection of health should focus on proper eating habits and regular physical activity, as they prevent chronic non-communicable diseases that result from behavioral and non-modifiable factors. These pillars, when balanced, prevent the onset of diseases and health problems, in addition to promoting quality of life ^10^. 

In view of this, studies focused on a situational diagnosis of health literacy are important to raise awareness about the practice and maintenance of healthy eating habits in the university environment, therefore improving the quality life of students. In this context, the present study aimed to assess the knowledge of health students about healthy eating (HE) and the factors associated with the university environment.

**KEY MESSAGES**

Motivation for the study: it is important to assess the level of knowledge about healthy eating in university students as this allows raising awareness about the practice and maintenance of healthy eating habits.

Main findings: most university students from the nine health careers had insufficient knowledge about healthy eating. The highest proportion of students with sufficient knowledge were found in the career of nutrition.

Implications: there is a need for projects at the university level that encompass the triad of psychology, food, and body; which would improve healthy eating habits in university students.

## MATERIALS AND METHODS

### Study design and location

This was a cross-sectional study that originated from an umbrella project called “Health Promotion in the young population: what is the role of the University?”, which was carried out in a university located in the city of Fortaleza, Ceará, Brazil. Data was collected from April to November 2017.

The university we selected for the study offers 40 undergraduate courses, has a staff of more than 1100 professors and has graduated approximately 100,000 undergraduate students, in addition to another 7000 postgraduate students ^11^.

### Study sample

The study included 512 university students (≥18 years), regardless of sex, who were enrolled, stratified, and equally distributed in nine undergraduate health courses (Physical Education, Nursing, Pharmacy, Physiotherapy, Speech Therapy, Medicine, Nutrition, Dentistry and Psychology), who were in semesters of the first or final year of the degree. Students with visual and physical impairments as well as pregnant women were excluded (participant self-report) due to the lack of adaptability of the collection instruments.

For the calculation of the minimum sample size, we considered the finite population of university students (n=161,199) enrolled in Fortaleza in 2015 ^12^, a prevalence of 53.8% of overweight in the young Brazilian population ^13^, a confidence interval of 95%, maximum error of 5% and a non-response rate of 10%, through the formula: n= [DE*Np(1-p) ]/ [ (d^2^/Z^2^_1-α/2_*(N-1)+p*(1-p) ], where DE=design effect, N=finite population size, p=frequency of the studied deviation, d=absolute precision and Z=constant.

The minimum sample size calculated was 458 university students; however, due to the need for an equal distribution between courses and semesters, we increased the number of participants, resulting in a non-probabilistic sample of 57 students from Physical Education, 57 from Nursing, 58 from Pharmacy, 58 from Physiotherapy, 46 from Speech Therapy, 58 from Medicine, 60 from Nutrition, 59 from Dentistry and 59 from Psychology.

The selection of university students was stratified by course and semester. First, we selected the disciplines of the first and last years of the nine health courses. Then, different days of the week and different times were randomly selected to evaluate each course. The students of the selected courses were directly invited to participate in the research before or after class.

### Data collection instruments and procedures

Data collection was carried out in two stages. In the first stage, two collection instruments were used: the University Health Promotion Assessment Instrument (Instrumento de Avaliação da Promoção da Saúde Na Universidade - IAPSU) and the International Physical Activity Questionnaire (IPAQ). The IAPSU is a self-administered questionnaire, with 41 items distributed in demographic and socioeconomic characteristics and five domains: 1) physical activity, 2) diet, 3) environmental factors, 4) psychosocial factors and alcohol and drug use, and 5) Integrative and Complementary Practices ^14^. 

The IAPSU variables that were analyzed in this study were: sociodemographic (gender, age in years, parental education and paid work), academic (academic year and semester), physical activity domain (participation in physical and/or recreational activities promoted by the university), eating domain (participation in activities related to healthy eating at the university, knowledge about healthy eating, healthy food supply and comfortable eating environment) and psychosocial factors domain (participation in self-esteem/self-awareness activities and health-promoting behaviors promoted by the university). This instrument has a reliability above 0.8 in all five domains. However, there are no validity data due to the absence of questionnaires for comparison ^14^.

The outcome variable, knowledge about HE, was obtained by adding the answers (yes=1; no=0) to five questions referring to the number of daily meals, consumption of fat-free foods, fiber intake, water intake and inclusion of proteins, calories, vitamins, and minerals in meals. We used a dichotomous classification, considering sufficient knowledge as having all answers correct (totaling 5 points) and insufficient knowledge as having at least one incorrect answer (score below 5 points).

The short version of the International Physical Activity Questionnaire (IPAQ), validated for Portuguese by Graig *et al*. ^(^^15^, evaluates the level of physical activity with eight questions that estimate the time spent by week and the intensity of physical activity at different moments: work, transportation, domestic activities, and leisure. This instrument presents good properties for monitoring the physical activity levels of adults (18-65 years) in different contexts, with a reliability of 0.91 and validity between 0.48 and 0.75 for different assessment methods. In this study, the level of physical activity was classified as: active (active and very active with ≥ 150 min/week) and not active (sedentary and insufficiently active with < 150 min/week).

The second stage of data collection comprised the anthropometric evaluation, which included the measurement of body weight, height, and abdominal perimeter. A portable digital scale (Plenna® brand) with a capacity of 150 kg and sensitivity of 100 g was used to measure body weight. The participant stood in the center of the equipment, dressed in as little clothing as possible, barefoot, with the body erect, arms along the body, head up and weight evenly distributed on both feet ^15^. If the participant was wearing jeans, 500 grams were removed from the measured weight. 

Height was measured in meters with a portable vertical stadiometer (Sanny® brand), which had a capacity of 2.11 m and a sensitivity of 0.5 cm. The measurement was carried out with the participant standing upright, with arms relaxed along the body, barefoot and with the head raised, positioned in the Frankfurt plane (inferior margin of the orbital opening and superior margin of the external auditory meatus on the same horizontal line) and free of accessories. The participant was positioned in such a way that the buttocks and the nape (occipital region) touched the stadiometer ^16^.

Body mass index (BMI) was calculated by dividing weight (in kilograms) by height (in meters) squared. BMI was classified dichotomously according to excess weight as no (< 25.0 kg/m2) and yes (≥ 25.0 kg/m^2^) ^17^.

We used a non-stretchable anthropometric tape (Sanny brand), measured in centimeters, to measure the abdominal perimeter (AP). The participant was standing with arms crossed over the chest and the measurement was taken at the end of a normal exhalation. When it was not possible to visualize narrower areas, the measurement was taken at the midpoint between the lower costal margin (10th rib) and the iliac crest, this variable was classified as: normal and altered (> 88 cm for women and > 102 cm for men ^18^.

Data was collected by a team of researchers comprised of previously trained health professionals and fellows, who followed a manual containing the description of all the stages of the study and the standardization of the collection methods in order to minimize errors during data collection.

### Data analysis

Descriptive and inferential statistics were applied using SPSS Statistics version 23.0. Variables were described by relative (%) and absolute (n) frequencies. For inferential analysis, some variables were reorganized: categorized age (< 25 years; ≥ 25 years), paternal and maternal schooling (≤ 8 years and > 8 years of study). During the bivariate analysis, the association between the outcome variable (knowledge about AS) and those of interest (sociodemographic, academic, factors associated with the university environment, level of physical activity, BMI and AP) was evaluated using the chi-square test and crude prevalence ratio (PR) and their respective 95% confidence intervals (95% CI).

Subsequently, we carried out a multivariate regression analysis using stepwise backward logistic regression with the inclusion of variables that were significant up to <0.20 in the bivariate analysis, in order to control for possible confounding variables. Adjusted PRs and their respective confidence intervals were estimated with a significance level of 5% for the construction of the final model.

### Ethical aspects

This study was approved by the Ethics Committee of the institution (University of Fortaleza - UNIFOR, Ceará, Brazil, ruling no. 1.795.390). The participating university students signed the free and informed consent form, which contained the objectives, risks and benefits of the study.

## RESULTS

Regarding the sociodemographic characteristics of the sample, most participants were female (69.7%; n=357) and under 25 years of age (76.8%; n=393). As for maternal schooling, the results showed that 58.3% (n=298) of the participants had mothers with more than 8 years of schooling, whereas, for paternal schooling, more than half (n=255; 50.2) of the participants’ fathers had 8 years of schooling or less. When asked about current employment, 83.8% (n=429) of respondents reported that they had no occupation (Table 1).

**Table 1:** Description of sociodemographic and academic variables of university students in health careers. Fortaleza, Ceará, 2017.

Variables	n (%)
Sociodemographic	
Sex	
Men	155 (30.3)
Women	357 (69.7)
Age groups	
< 25 years	393 (76.8)
≥ 25 years	119 (23.2)
Maternal schooling (years of schooling) (n=511)	
≤ 8 years	213 (41.7)
> 8 years	298 (58.3)
Paternal schooling (years of schooling) (n=508)	
≤ 8 years	255 (50.2)
> 8 years	253 (49.8)
Paid job	
No	429 (83.8)
Yes	83 (16.2)
Academics	
Undergraduate career	
Physical education	57 (11.1)
Nursing	57 (11.1)
Pharmacy	58 (11.3)
Physiotherapy	58 (11.3)
Language therapy	46 (9.0)
Medicine	58 (11.3)
Nutrition	60 (11.7)
Odontology	59 (11.5)
Psychology	59 (11.5)
Semester	
First year	253 (49.4)
Last	259 (50.6)

We found that most university students from the nine health courses had insufficient knowledge about healthy eating (71.9%; n=368). Most participants (15.3%; n=22) with sufficient level of knowledge were from the nutrition course, followed by those in the physical education course (12.5%; n=18) and students from the psychology and pharmacy courses, both with the same figures (11.8%; n=17). Finally, the fewest number of students with sufficient knowledge was found in medicine (8.3%; n=12;). During the bivariate analysis, we found that the sociodemographic and academic variables did not present a significant association with knowledge about HE (Table 2).

**Table 2:** Bivariate analysis between knowledge about healthy eating (HE) and sociodemographic and academic variables of university students in health careers. Fortaleza, Ceará, 2017.

Variables	Knowledge about HE		Crude PR (95% CI)	p-value
	Insufficient n (%)	Sufficient n (%)		
Sociodemographic				
Sex				0.733
Men	113 (30.7)	42 (29.2)	1	
Women	255 (69.3)	102 (70.8)	1.07 (0.70-1.64)	
Age group				0.556
< 25 years	285 (77.4)	108 (75.0)	1	
≥ 25 years	83 (22.6)	36 (25.0)	1.14 (0.73-1.79)	
Maternal schooling (years of schooling)				0.838
≤ 8 years	154 (42.0)	59 (41.0)	1	
> 8 years	213 (58.0)	85 (59.0)	1.04 (0.70-1.54)	
Paternal schooling (years of schooling)				0.887
≤ 8 years	183 (50.0)	74 (50.7)	1	
> 8 years	183 (50.0)	70 (49.3)	0.97 (0.66-1.43)	
Paid job				0.373
No	305 (82.9)	124 (86.1)	1	
Yes	63 (17.1)	20 (13.9)	0.78 (0.45-1.34)	
Academics				
Undergraduate course				0.588
Physical education	39 (10.6)	18 (12.5)	1	
Nursing	43 (11.7)	14 (9.7)	0.70 (0.31-1.60)	
Pharmacy	41 (11.1)	17 (11.8)	0.89 (0.40-1.98)	
Physiotherapy	44 (12.0)	14 (9.7)	0.68 (0.30-1.56)	
Language therapy	30 (8.2)	16 (11.1)	1.15 (0.50-2.63)	
Medicine	46 (12.5)	12 (8.3)	0.56 (0.24-1.31)	
Nutrition	38 (10.3)	22 (15.3)	1.25 (0.58-2.70)	
Odontology	45 (12.2)	14 (9.7)	0.67 (0.29-1.53)	
Psychology	42 (11.4)	17 (11.8)	0.87 (0.39-1.93)	
Semester				0.717
First year	180 (48.9)	73 (50.7)	1	
Last year	188 (51.1)	71 (49.3)	0.93 (0.63-1.36)	

PR: Prevalence Ratio; 95% CI: 95% Confidence Interval.

Regarding factors related to the university environment, most students with sufficient knowledge of HE participated in recreational activities promoted by the university (PR=1.77; p=0.049) and in healthy eating activities at the university (PR=1.87; p=0.013) (Table 3). We also found that the proportion of students with sufficient knowledge of HE was higher in students who were actively engaged in physical activity than in those who were overweight (PR=1.51; p=0.037) (Table 4).

**Table 3:** Bivariate analysis between knowledge about healthy eating (HE) of students and factors associated with the environment and activities promoted by the university. Fortaleza, Ceará, 2017.

Factors associated with the university environment	Knowledge about HE		Crude PR (95% CI)	p-value
	Insufficient n (%)	Sufficient n (%)		
Participation in physical activities promoted by the university				0.114^a^
No	328 (89.1)	121 (84.0)	1	
Yes	40 (10.9)	23 (16.0)	1.55 (0.89-2.71)	
Participation in recreational activities promoted by the university				0.049^a,b^
No	334 (90.8)	122 (84.7)	1	
Yes	34 (9.2)	22 (15.3)	1.77 (0.99-3.14)	
Participation in healthy eating activities at the university.				0.013^a,b^
No	321 (87.2)	113 (78.5)	1	
Yes	47 (12.8)	31 (21.5)	1.87 (1.13-3.09)	
Offering of healthy food by the university restaurants and/or cafeteria.				0.914
No	189 (51.9)	74 (51.4)	1	
Yes	175 (48.1)	70 (48.6)	1.02 (0.69-1.50)	
Is there a comfortable environment for eating?				0.683
No	129 (35.2)	48 (33.3)	1	
Yes	237 (64.8)	96 (66.7)	1.08 (0.72-1.63)	
Participation in activities about self-esteem and self-knowledge				0.163^a^
No	275 (74.7)	116 (80.6)	1	
Yes	93 (25.3)	28 (19.4)	0.71 (0.44-1.14)	
Participation in activities that encourage health-promoting behaviors				0.566
No	204 (55.6)	76 (52.8)	1	
Yes	163 (44.4)	68 (47.2)	1.12 (0.76-1.64)	

HE: Healthy Eating; PR: Prevalence Ratio; 95% CI: 95% Confidence Interval.

a variables selected for the regression model (Table 5); ^b^ p<0.05, by chi-square.

**Table 4:** Bivariate analysis between knowledge about healthy eating (HE) and the level of physical activity, excess weight, and abdominal perimeter of university students in health careers. Fortaleza, Ceará, 2017.

Variables	Knowledge about HE		Crude PR (95% CI)	p-value
	Insufficient n (%)	Sufficient n (%)		
Level of physical activity				0.174^a^
Non-active	231 (62.8)	81 (56.3)	1	
Active	137 (37.2)	63 (43.7)	1.31(0.88-1.93)	
Excess weight				0.037^a,b^
No	241 (65.5)	80 (55.6)	1	
Yes	127 (34.5)	64 (44.4)	1.51 (1.02 -2.24)	
Abdominal perimeter				0.098
Normal	333 (90.5)	123 (85.4)	1	
Altered	35 (9.5)	21 (14.6)	1.62 (0.91-2.89)	

HE: Healthy Eating; PR: Prevalence Ratio; 95% CI: 95% Confidence Interval.

a variable selected for the regression model (Table 5); ^b^ p<0.05, by chi-square.

Multivariate analysis confirmed that the following factors influence knowledge about healthy eating: participation in healthy eating activities (p=0.012; PR=1.94), participation in self-esteem and self-knowledge activities (p=0.046; PR=0.59) and being overweight (p=0.036; PR=1.53) (Table 5).

**Table 5:** Multivariate analysis of knowledge about healthy eating (HE) of health students and associated factors. Fortaleza, Ceará, 2017.

Variables	Knowledge about HE Adjusted PR (95% CI)	p-value
Participation in physical activities promoted by the university (yes)	1.12 (0.58 - 2.15)	0.719
Participation in recreational activities promoted by the university (yes)	1.67 (0.85 - 3.26)	0.133
Participation in healthy eating activities at the university (yes)	1.85 (1.09 - 3.13)	0.022^a^
Participation in activities that address self-esteem and self-knowledge (yes)	0.57 (0.34 - 0.95)	0.034^a^
Physical activity level (active)	0.74 (0.47 - 1.17)	0.206
Excess weight (yes)	1.52 (1.01 - 2.27)	0.040^a^

HE: Healthy Eating; PR: Prevalence Ratio; 95% CI: 95% Confidence Interval.

a p<0.05, by logistic regression. χ² of the model: 19.005.

## DISCUSSION

This study aimed to evaluate knowledge about HE in future health professionals. To this end, we evaluated the knowledge of university students entering and concluding a health course. We identified that the prevalence of insufficient knowledge about HE was high among the students. Having knowledge about HE, as well as understanding its importance, are the first steps towards changes in eating behavior. Therefore, assessing nutritional knowledge is necessary and important, as the level of such knowledge is related to people’s eating habits. By having greater nutritional knowledge, people will be able to adopt changes in order to improve their quality of life ^19^.

Our results showed that nutrition and physical education students had higher prevalence of adequate knowledge about HE. In agreement with our findings, a study conducted in the Southeast region of Brazil found that 46.1% of Nutrition students had a very good level of knowledge about healthy eating ^20^. Another study in the same region of Brazil showed that Physical Education university students knew the composition of their meals ^21^. Both studies pointed out that these results are related to the academic training and the nutrition-related contents addressed in the courses, in addition to highlighting the benefits of adequate knowledge about HE, such as increasing well-being and improving the quality of life of their future patients ^20^^,^^21^.

However, academic training in Nutrition and the inclusion of related subjects in health careers may still be insufficient to promote healthy behaviors, since inadequate eating habits are still being reported for university students, such as changing meals for snacks, consumption of sweets and fast food; added to the negative lifestyle and the lack of physical exercise ^7^.

This scenario is also the subject of research in different countries. A study with Chinese university students demonstrated the relationship between satisfactory knowledge and healthy attitudes and behaviors. These findings reinforce the importance of educational interventions to improve food-related knowledge in out-of-home settings, which is necessary to promote healthy eating behaviors among students ^22^.

Similarly, a study conducted on U.S. university students identified a relationship between greater nutritional knowledge and lower consumption of unhealthy fats. According to this study, nutritional education is a potential tool for health campaigns to promote healthy eating patterns in this population ^23^.

College years represent an important period for determining healthy habits and behaviors that promote wellness, proper nutrition, weight control, stress management, and regular exercise ^24^. In addition to that, a study conducted in Malaysian university students found that a good level of knowledge about healthy eating was more frequent among overweight university students. However, the study did not delve into the possible cause of the relationship between these variables ^25^.

Another study on Brazilian university students (Bahia) concluded that most students reported having access to information on healthy eating habits and lifestyle; however, most of them confirmed that they did not eat adequately, thus demonstrating that having access to information does not guarantee an adequate quality of life. Besides, most of the university students surveyed reported having a sedentary lifestyle, and not did not any physical activity, which makes students more susceptible to diseases associated with poor lifestyle habits ^26^.

Regarding the role of the university in promoting healthy eating habits, a survey of university students showed that knowledge about healthy eating and its practice is still insufficient, so a “strategic education for health and behavioral change” was recommended to motivate students to adopt a healthier diet ^27^. Accordingly, another study on university students promoted changes in food-related beliefs and behaviors, such as reducing the consumption of meat or avoiding industrialized foods, proving that educational actions and campaigns can help to promote changes ^28^.

In view of our results, we recommend the promotion of projects at the university level that cover the triad: psychology, nutrition, and body; thus, involving psychology, nutrition, and physical education courses, with the aim of guiding students who seek help in these areas. This would improve health of a great number of university students, which would lead to better academic performance, since those three areas intervene directly in cognitive development and, consequently, in the learning process.

Our study has some limitations. Mainly, the study design does not allow a cause-effect analysis, the sample is not probabilistic and the sample size is insufficient to identify differences between the careers (although the study presents an adequate level of statistical significance, effect size and power, evidenced by the sample size calculation). Other limitations are the non-use of a validated instrument to assess knowledge about HE and the risk of misclassifying people with sufficient knowledge, due to the arbitrary cut-off point. In addition, recall bias could have influenced the participant’s answers. Furthermore, variables related to social, personal, and cultural factors were not assessed.

Our results show a low percentage of health students with knowledge about healthy eating. However, participation in activities related to healthy eating, self-esteem and self-knowledge at the university improved this knowledge. We recommend the development of projects at the university level that encompass the psychological, nutritional and body triad, thus involving all health careers with the aim of improving the health and quality of life of university students.

## References

[1] Brasil, Ministerio de Salud (2014). Guía Alimentaria para la Población Brasileña promoviendo una alimentación saludable. Normas y manuales técnicos.

[2] Rodrigues RM, Souza A de M, Bezerra I N, Pereira RA, Yokoo EM, Sichieri R Most consumed foods in Brazil: evolution between 2008-2009 and 2017-2018. Revista De Saúde Pública.

[3] Oliveira JS, Santos DO, Rodrigues SJM, De Oliveira CC, Souza ALC (2018). Avaliação do perfil sociodemográfico, nutricional e alimentar de estudantes de nutrição de uma universidade pública em Lagarto-SE. Revista Da Associação Brasileira De Nutrição - RASBRAN.

[4] Souza RK, Backes V (2020). Autopercepção do consumo alimentar e adesão aos Dez Passos para Alimentação Saudável entre universitários de Porto Alegre, Brasil. Cien Salud Colet.

[5] Roy R, Kelly B, Rangan A, Allman-Farinelli M (2015). Food Environment Interventions to Improve the Dietary Behavior of Young Adults in Tertiary Education Settings A Systematic Literature Review. J Acad Nutr Diet.

[6] Barbosa BCR (2020). Práticas alimentares de estudantes universitários da área da saúde, de acordo com as recomendações do Guia Alimentar para a População Brasileira. Deméter.

[7] Rodrigues WP (2019). Avaliação de hábitos alimentares de universitários em Paripiranga-BA. Electronic Journal Collection Health.

[8] Bernardo GL (2017). Food intake of university students. Rev Nutri.

[9] Lorenzo Diputado (2016). Estado nutricionale hábitos alimentares de universitários. 2016. Rev Segur Alimento Nutri.

[10] Sato T (2017). Doenças crônicas não transmissíveis em usuários de Unidades de Saúde da Família - prevalência, perfil demográfico, utilização de serviços de saúde e necessidades clínicas. Rev Bras Ciênc Saúde.

[11] Fundação Edson Queiroz - Universidade de Fortaleza (2021). Fortaleza: Universidade de Fortaleza.

[12] Brasil (2017). Anuario de Ceará. Censo de Educación Superior - datos generales.

[13] Brasil, Ministerio de Salud, Departamento de Vigilancia Sanitaria (2019). Departamento de Análisis de Salud y Vigilancia de Enfermedades no Transmisibles. Vigitel Brasil 2018 - Vigilância de Fatores de Risco e Proteção para Doenças Crônicas por Inquérito Telefônico.

[14] Catrib AMF (2015). Desarrollo y Reproducibilidad del Instrumento de Evaluación de la Promoción de la Salud en la Universidad - IAPSU (Herramienta de Evaluación para la Promoción de la Salud en la Universidad). Rev Bras Promoç Saúde.

[15] Craig CL (2002). International Physical Activity Questionnaire: 12-Country Reliability and Validity. Medicine & Science in Sports & Exercise.

[16] BRASIL (2020). Encuesta de presupuestos familiares 2017-2018. Instituto Brasileño de Geografía y Estadística IBGE, Coordinación de Trabajo y Renta.

[17] Araujo MLD (2018). Precisão do IMC em diagnosticar o excesso de gordura corporal avaliada pela bioimpedância elétrica em universitários. Hospital Dietético Nutr Clín.

[18] Sociedad Brasileña de Diabetes (2020). https://diabetes.org.br/.

[19] Barbosa LB (2016). Estudos de avaliação do conhecimento nutricional de adultos uma revisão sistemática. Ciencias de la Salud Pública.

[20] Silveira MG (2019). Conhecimentos de acadêmicos de Nutrição sobre alimentação saudável e Nutrição Esportiva. RBNE - Revista Brasileña de Nutrición Deportiva.

[21] Oliveira JS, Santos DO, Rodrigues SJM, De Oliveira CC, Souza ALC (2018). Avaliação do perfil sociodemográfico, nutricional e alimentar de estudantes de nutrição de uma universidade pública em Lagarto-SE. Revista Da Associação Brasileira De Nutrição - RASBRAN.

[22] Hu P Knowledge, Attitude, and Behaviors Related to Eating Out among University Students in China. Int J Environ Res Salud Pública.

[23] Yahia N, Brown CA, Rapley M, Chung M (2016). Level of nutrition knowledge and its association with fat consumption among college students. BMC Public Health.

[24] Çetinkaya S, Sert H (2021). Healthy lifestyle behaviors of university students and related factors. Actas Paul Enferm.

[25] Hassan MR (2015). Knowledge, Attitude and Practice of Healthy Eating and Associated Factors among University Students in Selangor, Malaysia. Pak J Nutr.

[26] Souza M, Souza F (2016). Avaliação dos hábitos alimentares dos Universitários de uma Instituição Privada de Ensino Superior no interior da Bahia. Revista de Psicología.

[27] Dada SO, Oyewole OE, Desmennu AT (2021). Knowledge as Determinant of Healthy-Eating Among Male Postgraduate Public Health Students in a Nigerian Tertiary Institution. International Quarterly of Community Health Education.

[28] Campbell-Arvai V (2015). Food-related environmental beliefs and behaviours among university undergraduates: A mixed-methods study. International Journal of Sustainability in Higher Education.

