# Supplementary material for: Health students’ knowledge about healthy eating and factors associated with the university environment
Source: Rev Peru Med Exp Salud Publica. 2022 Dec 29;39(4):425–33. doi: 10.17843/rpmesp.2022.394.11349 (PMC11397587; doi:10.17843/rpmesp.2022.394.11349)
Supplement: Translation

# Translation: Conocimiento de estudiantes de salud sobre alimentación saludable y factores asociados al entorno universitario

**Authors:** Isabelle Cerqueira Sousa, Ana Maria Fontenelle Catrib, Natasha Teixeira Medeiros, Carla Christina Pereira da Silva Godinho, Antonio Augusto Ferreira Carioca, Gabriela Pessoa Marinho Holanda, Ilana Nogueira Bezerra, Ana Paula Vasconcellos Abdon

**Affiliations:** 1 Programa de Posgrado en Salud Colectiva (PPGSC), Universidad de Fortaleza (UNIFOR), Fortaleza, Ceará, Brasil.; 2 Universidad Federal del Delta do Parnaíba (UFDPar), Parnaíba, Piauí, Brasil; 3 Universidad de Fortaleza (UNIFOR), Fortaleza, Ceará, Brasil; 4 Programa de Posgrado en Salud Colectiva, Universidad Estatal de Ceará (UECE), Fortaleza, Ceará, Brasil

**Keywords:** Alimentación Saludable, Universidad, Promoción de la salud

## Abstract

**Objetivo.:**

Evaluar los conocimientos sobre alimentación saludable (AS) de los estudiantes del área de la salud y los factores asociados al entorno universitario.

**Materiales y métodos.:**

Se trata de un estudio transversal con 512 estudiantes universitarios (≥ 18 años) matriculados en nueve carreras de grado en salud. Se llevó a cabo de abril a noviembre de 2017. Se aplicaron el Instrumento Evaluativo de la promoción de la salud en la universidad y el Cuestionario Internacional de Actividad Física. Además, se midió el peso, talla y perímetro abdominal. Se realizaron análisis bivariados y multivariados utilizando SPSS versión 23.0.

**Resultados.:**

Se encontró que la mayoría de los estudiantes universitarios de las nueve carreras de salud tenían conocimientos insuficientes sobre alimentación saludable (71,9%; n=368). Sin embargo, la mayor proporción de estudiantes con conocimientos suficientes se encontraron en el área de nutrición (15,3%; n=22), seguidos de aquellos del área de educación física (12,5%; n=18). El menor porcentaje de estudiantes con conocimientos suficientes se encontró en la carrera de medicina (8,3%; n=12). En el análisis multivariado se evidenció que el conocimiento suficiente sobre alimentación saludable estaba relacionado con la participación en actividades de alimentación saludable (p=0,012; RP=1,94), participación en actividades que aborden la autoestima y el autoconocimiento (p=0,046; RP=0,59) y con tener exceso de peso (p=0,036; RP=1,53).

**Conclusión.:**

Se evidenció un bajo porcentaje de estudiantes de salud con conocimientos suficientes sobre alimentación saludable. Sin embargo, la participación en actividades de alimentación saludable, autoestima y autoconocimiento en la universidad logró mejorar el nivel de conocimiento. Se recomienda el desarrollo de proyectos universitarios que abarquen la tríada psicológica, alimentaria y corporal, involucrando así todas las carreras de salud, con el objetivo de mejorar la salud y la calidad de vida de los universitarios.

## INTRODUCCIÓN

La alimentación adecuada y saludable es un derecho humano, que debe estar alineado con los aspectos biológicos y sociales del individuo, de acuerdo con sus necesidades dietéticas especiales, y con base en el referente cultural de la región. Debe ser accesible en los aspectos físico y económico, armónico en cantidad y calidad, atendiendo a los principios de variedad, equilibrio, moderación y placer, además de basarse en prácticas de producción adecuadas y sostenibles ^1^.

La Encuesta de Presupuesto Familiar (*Pesquisa de Orçamentos Familiares* - POF) analizó el consumo de alimentos de la población brasileña adulta entre 2017 y 2018 y constató que hubo una reducción, en comparación con la POF realizada en la década anterior, en el consumo de arroz (OR=0,38 para hombres; OR=0,47 para mujeres) y frijoles (OR=0,32 para hombres; 0,41 para mujeres). Estos dos alimentos representan una combinación perfecta de aminoácidos, siendo parte de la alimentación tradicional en la cultura brasileña. Además, hubo un aumento en el consumo de sándwiches (OR=3,01 para hombres; OR=3,01 para mujeres) en todas las regiones del país y en todas las clases sociales, con una reducción en el consumo de frutas (OR=0,52 para hombres; OR=0,63 para mujeres) ^2^.

Los hábitos alimentarios de los universitarios cambian cuando ingresan a la universidad. La rutina estudiantil muchas veces puede influir negativamente en la alimentación, ya que una mayor cantidad de comidas se realizan fuera de casa, lo que ocasiona que los estudiantes dependan de establecimientos comerciales en la universidad o cercanos a ella^ (^ ^3^. Además, solo el 7% de los estudiantes universitarios planifican el tiempo de sus comidas a lo largo del día ^4^, aunque este sea uno de los diez pasos establecidos en la Guía Alimentaria de la Población Brasileña para una alimentación saludable ^1^.

El ambiente alimentario universitario puede ser un factor de exposición a enfermedades crónicas no transmisibles en adultos jóvenes, ya que los alimentos y bebidas no saludables son más accesibles. Según una revisión sistemática, las universidades de Estados Unidos y de la Unión Europea han advertido la importancia de esta cuestión y han puesto en marcha intervenciones para mejorar el entorno alimentario. Algunas de estas intervenciones se centraron en directrices sobre el etiquetado de alimentos, el aumento de la disponibilidad de alimentos saludables y la disminución del tamaño de la porción de alimentos no saludables ^5^. Estas acciones muestran la capacidad de las instituciones de educación superior para trabajar a favor de la promoción de la salud, especialmente la alimentaria.

También se evidencia que el hecho de cursar una carrera en el área de la salud no es un factor protector para tener una alimentación más saludable, siendo comunes las conductas alimentarias poco saludables, con alto consumo de comida rápida, dulces, refrescos y bebidas alcohólicas, así como el bajo consumo de frutas, verduras, pescado, cereales integrales y legumbres. Por lo tanto, independientemente del curso de pregrado, el consumo de alimentos por parte de los estudiantes universitarios se caracterizó como inadecuado ^6^ ^,^ ^7^ ^,^ ^8^ ^,^ ^9^.

Además, es posible evidenciar el creciente consumo de productos industrializados, especialmente dirigidos el público adulto y universitario; la mala comprensión de las etiquetas de los productos o incluso no leerlas también es un dato importante, por lo que es necesario tener una mirada más crítica en la elección de los alimentos ^7^.

Las actitudes fundamentales para la promoción y protección de la salud deben centrarse en hábitos alimentarios adecuados y prácticas regulares de actividad física, ya que previenen enfermedades crónicas no transmisibles, resultantes de factores conductuales y no modificables. Estos pilares, cuando están equilibrados, previenen la aparición de enfermedades y problemas de salud, además de promover la calidad de vida ^10^.

Frente a esto, los estudios enfocados a realizar un diagnóstico situacional sobre los conocimientos en salud son importantes para sensibilizar sobre la práctica y mantenimiento de una alimentación saludable en el ámbito universitario con miras a contribuir al estilo de vida de los estudiantes. En ese contexto, el presente estudio tuvo como objetivo evaluar los conocimientos sobre alimentación saludable (AS) de los estudiantes del área de la salud y los factores asociados al entorno universitario.

**MENSAJES CLAVE**

Motivación para realizar el estudio: Es importante evaluar el nivel de conocimientos acerca de alimentación saludable en estudiantes universitarios ya que esto permite sensibilizar sobre la práctica y mantenimiento de hábitos alimentarios saludables.

Principales hallazgos: La mayoría de los estudiantes universitarios de las nueve carreras de salud tenían conocimientos insuficientes sobre alimentación saludable. La mayor proporción de estudiantes con conocimientos suficientes se encontraron en el área de nutrición.

Implicancias: Es necesario promover proyectos en el ámbito universitario que abarquen la triada de psicología, alimentación y cuerpo; lo cual mejoraría los hábitos alimentarios saludables en estudiantes universitarios.

## MATERIALES Y MÉTODOS

### Diseño y lugar del estudio

Este fue un estudio transversal, originado en un proyecto paraguas denominado «Promoción de la Salud en la población joven: ¿cuál es el papel de la Universidad?», desarrollado en una universidad ubicada en la ciudad de Fortaleza, Ceará, Brasil. La recolección de datos se llevó a cabo de abril a noviembre de 2017.

La universidad seleccionada ofrece 40 cursos de pregrado, cuenta con un cuerpo docente de más de 1100 profesores y ha graduado aproximadamente 100 000 estudiantes de pregrado, además de otros 7000 de postgrado^ (^ ^11^.

### Muestra del estudio

Participaron del estudio 512 estudiantes universitarios (≥18 años), independientemente del sexo y matriculados en cursos de pregrado en salud, distribuidos de forma estratificada y equitativa en nueve cursos (Educación Física, Enfermería, Farmacia, Fisioterapia, Terapia del Lenguaje, Medicina, Nutrición, Odontología y Psicología) y por semestre académico (primer y último año de graduación). Los estudiantes con deficiencias visuales y físicas y las mujeres embarazadas fueron excluidos (autoinforme del participante) debido a la falta de adaptabilidad de los instrumentos de recolección.

Se realizó el cálculo del tamaño mínimo muestral considerando una población finita de universitarios (n=161 199) matriculados en Fortaleza en el año 2015^ (^ ^12^, una prevalencia de 53,8% de exceso de peso en la población joven brasileña^ (^ ^13^, un intervalo de confianza del 95%, error máximo del 5% y un índice de no respuesta del 10%, a través de la fórmula: n= [ED*Np(1-p)]/[(d^2^/Z^2^_1-α/2_*(N-1)+p*(1-p)], donde ED=efecto de diseño, N=tamaño de población finita, p=frecuencia de la desviación estudiada, d=precisión absoluta y Z=constante.

La cantidad mínima calculada fue de 458 universitarios, sin embargo, debido a la necesidad de una distribución equitativa entre cursos y semestres se incrementó el número de participantes, resultando en una muestra no probabilística de 57 estudiantes de la carrera de Educación Física, 57 de Enfermería, 58 de Farmacia, 58 de Fisioterapia, 46 de Terapia del Lenguaje, 58 de Medicina, 60 de Nutrición, 59 de Odontología y 59 de Psicología.

La selección de estudiantes universitarios se estratificó por curso y semestre. Primero se realizó la selección de las disciplinas de los primeros y últimos años de los nueve cursos del área de la salud. Luego, las asignaturas se definieron por sorteo en diferentes días de la semana y en diferentes horarios. Los estudiantes de las asignaturas seleccionadas fueron invitados a participar en la investigación mediante un enfoque directo antes o después de las clases. 

### Instrumentos y procedimientos de recopilación de datos

La recolección de datos se llevó a cabo en dos etapas. En la primera etapa, se utilizaron dos instrumentos de recolección: el Instrumento Evaluativo de la Promoción de la Salud en la Universidad (*Instrumento de Avaliação da Promoção da Saúde Na Universidade - IAPSU*) y el Cuestionario Internacional de Actividad Física (*International Physical Activity Questionnaire - IPAQ*). El IAPSU es un cuestionario autoadministrado, con 41 ítems distribuidos en características demográficas y socioeconómicas y cinco dominios: 1) actividad física, 2) alimentación, 3) factores ambientales, 4) factores psicosociales y consumo de alcohol y drogas, y 5) Prácticas Integrativas y Complementarias^ (^ ^14^. 

En este estudio se analizaron algunas variables del IAPSU: sociodemográficas (género, edad en años, educación de los padres y trabajo remunerado), académicas (curso y semestre académico), dominio de actividad física (participación en actividades físicas y/o recreativas promovidas por la universidad), dominio de alimentación (participación en actividades sobre alimentación saludable en la universidad, conocimiento sobre alimentación saludable, oferta de alimentos saludables y ambiente confortable para comer) y dominio de factores psicosociales (participación en actividades de autoestima/autoconocimiento y conductas promotoras de salud promovidas por la universidad). Este instrumento tiene una fiabilidad superior a 0,8 en los cinco dominios. Sin embargo, no existen valores de validez debido a la ausencia de cuestionarios para la comparación^ (^ ^14^.

La variable de resultado, conocimiento sobre AS, se obtuvo sumando las respuestas (sí=1; no=0) a cinco preguntas referentes a la cantidad de comidas diarias, consumo de alimentos libres de grasas, consumo de fibra, ingesta de agua e inclusión de proteínas, calorías, vitaminas y minerales en las comidas. Se estableció una clasificación dicotómica, considerando conocimiento suficiente como tener todas las respuestas correctas (sumando 5 puntos) y conocimiento insuficiente como al menos una respuesta incorrecta (puntaje inferior a 5 puntos).

La versión corta del Cuestionario Internacional de Actividad Física (IPAQ), validado para idioma portugués por Graig *et al*.^15^, determina el nivel de actividad física mediante ocho preguntas que estiman el tiempo dedicado semanalmente y la intensidad de la actividad física en diferentes momentos: trabajo, transporte, actividades domésticas y ocio. Este instrumento presenta buenas propiedades para monitorear los niveles de actividad física de adultos (18-65 años) en diversos contextos, teniendo confiabilidad de 0,91 y validez entre 0,48 a 0,75 para diferentes métodos de evaluación. En este estudio, el nivel de actividad física se clasificó en: activa (activa y muy activa con ≥ 150 min/semana) y no activa (sedentaria e insuficientemente activa con < 150 min/semana). 

En la segunda etapa se realizó la valoración antropométrica con la medición del peso corporal, talla y perímetro abdominal. Para medir el peso corporal se utilizó una balanza digital portátil (marca Plenna®), con capacidad de 150 kg y sensibilidad de 100g. El participante se paraba en el centro del equipo, vestido con la menor ropa posible, descalzo, con el cuerpo erguido, los brazos a lo largo del cuerpo, la cabeza en alto y el peso distribuido uniformemente en ambos pies^ (^ ^15^. Se eliminaron 500 gramos del peso medido si el participante vestía jeans.

La altura en metros se midió con un estadiómetro vertical portátil (marca Sanny®) con una capacidad de 2,11 m y una sensibilidad de 0,5 cm. Se midió con el participante de pie, erguido, con los brazos relajados a lo largo del cuerpo, descalzo y con la cabeza levantada, posicionado en el plano de Frankfurt (margen inferior de la apertura orbitaria y margen superior del meato auditivo externo en una misma línea horizontal) y libre de accesorios. El participante se posicionó rozando los glúteos y la nuca (región occipital) con el estadiómetro^ (^ ^16^.

El índice de masa corporal (IMC) se calculó dividiendo el peso (en kilogramos) por la altura (en metros) al cuadrado. El IMC se clasificó de manera dicotómica en función del exceso de peso como no (< 25,0 kg/m^2^) y sí (≥ 25,0 kg/m^2^) ^17^.

Para medir el perímetro abdominal (PA) se utilizó una cinta antropométrica no extensible (marca Sanny), medida en centímetros. El participante estaba de pie con los brazos cruzados sobre el pecho y la medida se tomaba al final de una espiración normal. Cuando no era posible visualizar zonas más estrechas, la medida se tomaba en el punto medio entre el margen costal inferior (10ª costilla) y la cresta ilíaca, esta variable se clasificó en: normal y alterada (> 88 cm para mujeres y > 102 cm para hombres^ (^ ^18^.

La recolección de datos fue realizada por un equipo de investigadores integrado por profesionales de la salud y becarios, previamente capacitados, que siguieron un manual conteniendo la descripción de todas las etapas del estudio y la estandarización de los métodos de recolección con el fin de minimizar errores durante la recolección de datos. 

### Análisis de los datos

Se utilizó estadística descriptiva e inferencial mediante el programa SPSS *Statistics* versión 23.0. Las variables se describieron mediante frecuencias relativas (%) y absolutas (n). Para el análisis inferencial, algunas variables fueron reorganizadas en: edad categorizada (< 25 años; ≥ 25 años), escolaridad paterna y materna (≤ 8 años y > 8 años de estudio). Durante el análisis bivariado se evaluó la asociación entre la variable de resultado (conocimiento sobre AS) y las de interés (sociodemográficas, académicas, factores asociados al entorno universitario, nivel de actividad física, IMC y PA) utilizando la prueba de chi-cuadrado y medidas de asociación de razón de prevalencia (RP) crudas y sus respectivos intervalos de confianza del 95% (IC 95%).

Posteriormente, se realizó un análisis de regresión multivariante mediante regresión logística por el método *stepwise backward* con la inclusión de variables que presentaron significancia hasta <0,20 en el análisis bivariado, con el fin de controlar posibles variables de confusión. Se estimaron las RP ajustadas y sus respectivos intervalos de confianza con un nivel de significancia del 5% para la construcción del modelo final.

### Aspectos éticos

Este estudio fue aprobado por el Comité de Ética de la institución (Universidad de Fortaleza - UNIFOR, Ceará, Brasil, dictamen nº 1.795.390). Los estudiantes universitarios participantes firmaron el formulario de consentimiento libre e informado, que contenía los objetivos, riesgos y beneficios del estudio.

## RESULTADOS

Con respecto a las características sociodemográficas de la muestra, se encontró mayor proporción de participantes de sexo femenino (69,7%; n=357), y menores de 25 años (76,8%; n=393). En cuanto a la escolaridad materna, los resultados mostraron que el 58,3% (n=298) de los participantes tenía madres con más de 8 años de escolaridad, mientras que, para la escolaridad paterna, más de la mitad (n=255; 50,2) de los padres de los participantes tenía 8 años de escolaridad o menos. Cuando se les preguntó sobre el trabajo actual, el 83,8% (n=429) de los encuestados respondió que no tenía ocupación (Tabla 1).

**Tabla 1:** Descripción de variables sociodemográficas y académicas de estudiantes universitarios del área de la salud. Fortaleza, Ceará, 2017.

Variables	n (%)
Sociodemográficas	
Sexo	
Masculino	155 (30,3)
Femenino	357 (69,7)
Grupo de edad	
< 25 años	393 (76,8)
≥ 25 años	119 (23,2)
Escolaridad materna (años de estudio) (n=511)	
≤ 8 años	213 (41,7)
> 8 años	298 (58,3)
Escolaridad paterna (años de estudio) (n=508)	
≤ 8 años	255 (50,2)
> 8 años	253 (49,8)
Trabajo pagado	
No	429 (83,8)
Sí	83 (16,2)
Académicas	
Carrera de pregrado	
Educación Física	57 (11,1)
Enfermería	57 (11,1)
Farmacia	58 (11,3)
Fisioterapia	58 (11,3)
Terapia del Lenguaje	46 (9,0)
Medicina	58 (11,3)
Nutrición	60 (11,7)
Odontología	59 (11,5)
Psicología	59 (11,5)
Semestre	
Primer año	253 (49,4)
Último año	259 (50,6)

Se observó que la mayoría de los estudiantes universitarios de los nueve cursos del área de la salud tenían conocimientos insuficientes sobre alimentación saludable (71,9%; n=368). En cuanto al conocimiento sobre AS, la mayor proporción (15,3%; n=22) de participantes con nivel suficiente de conocimiento pertenecían a la carrera de nutrición, seguidos de aquellos en la carrera de Educación física (12,5%; n=18) y de los estudiantes pertenecientes a las carreras de Psicología y Farmacia en (11,8%; n=17). Finalmente, la menor proporción de estudiantes con conocimientos suficientes se encontró en la carrera de medicina (8,3%; n=12;). Durante el análisis bivariado, fue posible verificar que las variables sociodemográficas y académicas no presentaron asociación significativa con el conocimiento sobre AS (Tabla 2).

**Tabla 2:** Análisis bivariado entre conocimiento sobre alimentación saludable (AS) con las variables sociodemográficas y académicas de los estudiantes universitarios del área de la salud. Fortaleza, Ceará, 2017.

Variables	Conocimiento sobre AS		RP crudo (IC95%)	p-valor
	Insuficiente n (%)	Suficiente n (%)		
Sociodemográficas				
Sexo				0,733
Masculino	113 (30,7)	42 (29,2)	1	
Femenino	255 (69,3)	102 (70,8)	1,07 (0,70-1,64)	
Grupo de edad				0,556
< 25 años	285 (77,4)	108 (75,0)	1	
≥ 25 años	83 (22,6)	36 (25,0)	1,14 (0,73-1,79)	
Escolaridad materna (años de estudio)				0,838
≤ 8 años	154 (42,0)	59 (41,0)	1	
> 8 años	213 (58,0)	85 (59,0)	1,04 (0,70-1,54)	
Escolaridad paterna (años de estudio)				0,887
≤ 8 años	183 (50,0)	74 (50,7)	1	
> 8 años	183 (50,0)	70 (49,3)	0,97 (0,66-1,43)	
Trabajo pagado				0,373
No	305 (82,9)	124 (86,1)	1	
Sí	63 (17,1)	20 (13,9)	0,78 (0,45-1,34)	
Académicas				
Curso de pregrado				0,588
Educación Física	39 (10,6)	18 (12,5)	1	
Enfermería	43 (11,7)	14 (9,7)	0,70 (0,31-1,60)	
Farmacia	41 (11,1)	17 (11,8)	0,89 (0,40-1,98)	
Fisioterapia	44 (12,0)	14 (9,7)	0,68 (0,30-1,56)	
Terapia del Lenguaje	30 (8,2)	16 (11,1)	1,15 (0,50-2,63)	
Medicina	46 (12,5)	12 (8,3)	0,56 (0,24-1,31)	
Nutrición	38 (10,3)	22 (15,3)	1,25 (0,58-2,70)	
Odontología	45 (12,2)	14 (9,7)	0,67 (0,29-1,53)	
Psicología	42 (11,4)	17 (11,8)	0,87 (0,39-1,93)	
Semestre				0,717
Primer año	180 (48,9)	73 (50,7)	1	
Último año	188 (51,1)	71 (49,3)	0,93 (0,63-1,36)	

RP: Razón de Prevalencia; IC95%: Intervalo de Confianza de 95%.

En cuanto a los factores relacionados con el entorno universitario, la mayor proporción de estudiantes con conocimientos suficientes sobre AS fueron aquellos que participaron en actividades recreativas promovidas por la universidad (RP=1,77; p=0,049) y en actividades de alimentación saludable en la universidad (RP=1,87; p=0,013) (Tabla 3). También se encontró que la proporción de estudiantes con conocimientos suficientes de AS fue mayor en los estudiantes que realizaban activamente actividad física que en aquellos que presentaban exceso de peso (RP=1,51; p=0,037) (Tabla 4).

**Tabla 3:** Análisis bivariado entre conocimiento sobre alimentación saludable (AS) de los estudiantes con los factores asociados al ambiente y actividades promovidas por la universidad. Fortaleza, Ceará, 2017.

Factores asociados con el entorno universitario	Conocimiento sobre AS		RP crudo (IC95%)	p-valor
	Insuficiente n (%)	Suficiente n (%)		
Participación en actividades físicas promovidas por la universidad				0,114^a^
No	328 (89,1)	121 (84,0)	1	
Sí	40 (10,9)	23 (16,0)	1,55 (0,89-2,71)	
Participación en actividades recreativas promovidas por la universidad				0,049^a,b^
No	334 (90,8)	122 (84,7)	1	
Sí	34 (9,2)	22 (15,3)	1,77 (0,99-3,14)	
Participación en actividades de alimentación saludable en la universidad				0,013^a,b^
No	321 (87,2)	113 (78,5)	1	
Sí	47 (12,8)	31 (21,5)	1,87 (1,13-3,09)	
Oferta de alimentos saludables por los restaurantes y/o cafetería de la universidad				0,914
No	189 (51,9)	74 (51,4)	1	
Sí	175 (48,1)	70 (48,6)	1,02 (0,69-1,50)	
Hay ambiente cómodo para hacer su alimentación				0,683
No	129 (35,2)	48 (33,3)	1	
Sí	237 (64,8)	96 (66,7)	1,08 (0,72-1,63)	
Participación en actividades que abordan autoestima y autoconocimiento				0,163^a^
No	275 (74,7)	116 (80,6)	1	
Sí	93 (25,3)	28 (19,4)	0,71 (0,44-1,14)	
Participación en actividades que incentivan comportamientos promotores de salud				0,566
No	204 (55,6)	76 (52,8)	1	
Sí	163 (44,4)	68 (47,2)	1,12 (0,76-1,64)	

AS: Alimentación Saludable; RP: Razón de Prevalencia; IC95%: Intervalo de Confianza de 95%.

a variables seleccionadas para el modelo de regresión (Tabla 5); ^b^ p<0,05, por chi-cuadrado.

**Tabla 4:** Análisis bivariado entre conocimiento sobre alimentación saludable (AS) con el nivel de actividad física, exceso de peso y perimetro abdominal de los universitarios del área de la salud. Fortaleza, Ceará, 2017.

Variables	Conocimiento sobre AS		RP crudo (IC95%)	p-valor
	Insuficiente n (%)	Suficiente n (%)		
Nivel de actividad física				
				0,174^a^
No activo	231 (62,8)	81 (56,3)	1	
Activo	137 (37,2)	63 (43,7)	1,31(0,88-1,93)	
Exceso de peso				0,037^a,b^
No	241 (65,5)	80 (55,6)	1	
Sí	127 (34,5)	64 (44,4)	1,51 (1,02 -2,24)	
Perimetro abdominal				0,098
Normal	333 (90,5)	123 (85,4)	1	
				
Alterada	35 (9,5)	21 (14,6)	1,62 (0,91-2,89)	

AS: Alimentación Saludable; RP: Razón de Prevalencia; IC95%: Intervalo de Confianza de 95%.

a variable seleccionada para el modelo de regresión (Tabla 5); ^b^ p<0,05, por chi-cuadrado.

El análisis multivariante confirmó la influencia de los siguientes factores sobre el conocimiento acerca de alimentación saludable: participación en actividades de alimentación saludable (p=0,012; RP=1,94), participación en actividades de autoestima y autoconocimiento (p=0,046; RP=0,59) y exceso de peso (p=0,036; RP=1,53) (Tabla 5).

**Tabla 5:** Análisis multivariado del conocimiento sobre alimentación saludable (AS) de los estudiantes del área de la salud y los demás factores asociados. Fortaleza, Ceará, 2017.

Variables	Conocimiento sobre AS RP ajustado (IC95%)	p-valor
Participación en actividades físicas promovidas por la universidad (sí)	1,12 (0,58 - 2,15)	0,719
Participación en actividades recreativas promovidas por la universidad (sí)	1,67 (0,85 - 3,26)	0,133
Participación en actividades de alimentación saludable en la universidad (sí)	1,85 (1,09 - 3,13)	0,022^a^
Participación en actividades que abordan autoestima y autoconocimiento (sí)	0,57 (0,34 - 0,95)	0,034^a^
Nivel de actividad física (activo)	0,74 (0,47 - 1,17)	0,206
Exceso de peso (sí)	1,52 (1,01 - 2,27)	0,040^a^

AS: Alimentación Saludable; RP: Razón de Prevalencia; IC95%: Intervalo de Confianza de 95%.

a p<0,05, por regresión logística. χ² del modelo: 19.005.

## DISCUSIÓN

Este estudio tuvo como objetivo evaluar los conocimientos sobre AS en los futuros profesionales de la salud. Para ello, se evaluó el porcentaje de estudiantes universitarios que ingresan y concluyen curso en el área de la salud en cuanto a conocimientos, luego de lo cual se identificó una gran prevalencia de conocimientos insuficientes sobre AS. Tener conocimiento sobre AS, así como entender su importancia son el primer paso hacia cambios en el comportamiento alimentario. Por lo tanto, evaluar el conocimiento nutricional es necesario e importante, ya que el nivel de ese conocimiento está relacionado con el comportamiento alimentario de las personas. A partir de un mayor conocimiento nutricional, podrán adoptar cambios que mejoren la calidad de vida^ (^ ^19^.

En la presente investigación, los estudiantes de nutrición y educación física tuvieron mayor prevalencia de conocimientos adecuados sobre AS. De acuerdo con estos hallazgos, un estudio realizado en la región Sudeste de Brasil constató que el 46,1% de los estudiantes de Nutrición mostraron muy buen nivel de conocimientos sobre alimentación saludable^ (^ ^20^. Otro estudio en la misma región del país mostró que los estudiantes universitarios de Educación Física conocían la composición de sus comidas^ (^ ^21^. Ambos estudios señalaron que estos resultados están relacionados con la formación académica y los contenidos relacionados a nutrición abordados en las asignaturas. Además de resaltar los beneficios del conocimiento adecuado sobre AS, como incrementar el bienestar y mejorar la calidad de vida de sus futuros pacientes^ (^ ^20^ ^,^ ^21^.

Sin embargo, la formación académica en Nutrición y la provisión de asignaturas afines en las carreras de salud pueden aún ser insuficientes para promover conductas saludables, ya que se observan hábitos alimentarios inadecuados, como el cambio de comidas por meriendas, consumo de dulces y comida rápida; sumado al estilo de vida negativo, respecto a la no realización de ejercicio físico por parte de los estudiantes universitarios^ (^ ^7^.

Este panorama también es foco de investigación en diferentes países. Un estudio con universitarios chinos demostró la relación entre conocimientos satisfactorios y actitudes y comportamientos saludables. Estos hallazgos refuerzan la importancia de las intervenciones educativas con respecto a mejorar los conocimientos relacionados con la alimentación en entornos fuera del hogar, los cuales son necesarios para promover conductas alimentarias saludables entre los estudiantes^ (^ ^22^.

De igual forma, un estudio realizado con estudiantes universitarios estadounidenses identificó una relación entre un mayor conocimiento nutricional y un menor consumo de grasas no saludables. Según este estudio, la educación nutricional es una herramienta potencial en las campañas de salud para promover patrones de alimentación saludables en esta población ^23^.

Ampliando estos hallazgos, se sabe que los años en la universidad representan un período importante para la determinación de hábitos y comportamientos saludables, que promuevan el bienestar, la nutrición adecuada, el control del peso, el manejo del estrés y el ejercicio regular^ (^ ^24^. Además de eso, un estudio realizado en estudiantes universitarios de Malasia descubrió que los conocimientos sobre alimentación saludable clasificados como buenos tenían porcentajes más altos en estudiantes universitarios con sobrepeso. Sin embargo, el estudio no profundizó en la posible causa de la relación entre estas variables^ (^ ^25^.

Otro estudio con estudiantes universitarios brasileños (Bahía) concluyó que la mayoría de los estudiantes reportaron tener acceso a información sobre hábitos de alimentación y estilo de vida saludables, sin embargo, la mayoría confirmó que no se alimentaba adecuadamente, demostrando así que el hecho de tener acceso a la información no garantiza una calidad de vida adecuada, además de que la mayoría de los universitarios encuestados reportaron tener una vida sedentaria, y no practicar ninguna actividad física, lo que hace que los estudiantes sean más susceptibles a enfermedades asociadas a malos hábitos de vida^ (^ ^26^.

En cuanto al papel de la universidad en el fomento de hábitos alimentarios saludables, una encuesta realizada a estudiantes universitarios mostró que el conocimiento sobre alimentación saludable y su práctica aún es insuficiente en la población estudiada, por lo que se recomendó una «educación estratégica para la salud y para el cambio de comportamiento» con el objetivo de motivar a los estudiantes a tener una alimentación más saludable^ (^ ^27^. En concordancia, otro estudio con universitarios promovió cambios en las creencias y comportamientos relacionados a alimentación, como reducir el consumo de carnes o evitar alimentos industrializados, comprobando que acciones y campañas educativas ayudan a promover modificaciones^ (^ ^28^.

En vista de los hallazgos de este estudio, se recomienda promover proyectos en el ámbito universitario que abarquen la tríada: psicología, alimentación y cuerpo; involucrando así cursos de psicología, nutrición y educación física, con el objetivo de orientar a los estudiantes que buscan ayuda en estas áreas. Esto mejoraría la salud en un mayor número de estudiantes universitarios, lo que ocasionaría un mejor rendimiento académico, ya que las tres áreas mencionadas intervienen directamente en el desarrollo cognitivo y, en consecuencia, en el proceso de aprendizaje.

Se deben considerar algunas limitaciones de nuestro estudio. Principalmente, el diseño de estudio no permite un análisis de causa-efecto, la muestra no es probabilística y el tamaño de muestra es insuficiente para identificar diferencias entre las carreras (aunque el estudio presenta un nivel adecuado de significación estadística, tamaño del efecto y poder, evidenciado por el cálculo del tamaño muestral). Otras limitaciones son: la no utilización de un instrumento validado para evaluar el conocimiento sobre AS y el riesgo de clasificar erróneamente a las personas con conocimientos suficientes, debido al punto de corte arbitrario. Además, también pudo haber presencia de sesgo de memoria al momento de realizar el llenado del formulario por parte del participante. Adicionalmente, las variables de factores sociales, personales y culturales no se evaluaron.

Los hallazgos de este estudio constataron que hubo un bajo porcentaje de estudiantes de salud con conocimientos sobre alimentación saludable. Sin embargo, la participación en actividades relacionadas a alimentación saludable, autoestima y autoconocimiento en la universidad promovieron una mejora en este conocimiento. Se recomienda el desarrollo de un proyecto a nivel universitario que abarque la tríada psicológica, alimentaria y corporal, involucrando así todas las carreras de salud con el objetivo de mejorar la salud y la calidad de vida de los universitarios.
